# Ketone Bodies Mediate Antiseizure Effects

**DOI:** 10.15844/pedneurbriefs-29-9-2

**Published:** 2015-09

**Authors:** Jena M. Krueger, Douglas R. Nordli

**Affiliations:** 1 Division of Neurology, Ann & Robert H. Lurie Children's Hospital of Chicago, Chicago, IL; 2 Departments of Pediatrics and Neurology, Northwestern University Feinberg School of Medicine, Chicago, IL

**Keywords:** Ketogenic Diet, Ketone Bodies, Epilepsy

## Abstract

Investigators from The Barrow Neurological Institute, Creighton University, University of Kentucky and the University of Calgary Faculty of Medicine investigated the effect of ketone bodies and the ketogenic diet on epileptic Kcna1-null mice.

Investigators from The Barrow Neurological Institute, Creighton University, University of Kentucky and the University of Calgary Faculty of Medicine investigated the effect of ketone bodies and the ketogenic diet on epileptic Kcna1-null mice. The Kcna1-null mouse is an animal model that represents human temporal lobe epilepsy. The mice were found to have a reduction in spontaneous recurrent seizures after treatment with the ketogenic diet when compared to Kcna1-null mice fed with a standard diet. In addition, seizure reduction in the Kcna1-null mice was also noted when the mice were administered ketones (beta-hydroxybutyrate) via implanted pump and fed an ad lib standard diet. They were found to have normal glucose levels. These results were replicated in vitro, on slice cultures prepared from Kcna1-null mice. Investigators also showed that the antiseizure properties of the ketogenic diet were mitigated when the mitochondrial permeability transition pore was activated. They found that ketone bodies were able to raise the threshold for calcium-induced mitochondrial permeability transition, which decreased activation of the mitochondrial permeability transition pore and thus hypothesized the blockade of this process is part of ketone body antiepileptic mechanism.

The investigators also found evidence of protective effects secondary to the ketogenic diet. Kcn1-null mice showed significant difficulties in spatial learning and memory when compared to wild type mice. Treatment with the ketogenic diet restored these deficits to levels seen in the wild type mice. This was re-demonstrated in vitro, as the Kcn1-null mice demonstrated impairment in CA1 hippocampal long term potentiation, which was restored by the ketogenic diet or administration of beta-hydroxybutyrate alone. [1]

COMMENTARY. The ketogenic diet is an important factor in the treatment of pediatric refractory epilepsy. A meta-analysis of available data suggests that a 50% reduction of seizures can been seen in up to 1/3 of patients on the diet [2]. In this paper the authors report evidence toward a new target in the ketogenic diet, which supports prior literature suggesting the ketogenic diet's effects rely on multiple mechanisms of action. Rho and Sankar suggested multiple mechanisms, including enhancement of the GABA system, direct effects from acetone and acetoacetate, increased energy substrates, decreased oxygen species or enhancing glutathione [3]. Similar to many effective antiseizure medications, the multifaceted pathways in the ketogenic diet lend themselves to a broad spectrum of seizure control, which can be more effective than singular therapies. Testing the ketogenic diet utilizing different mouse models yields new insight into the different mechanisms.

The authors also provide evidence ketone bodies may restore deficits in learning and memory. Cognitive dysfunction is common in pediatric epilepsy [4]. However, causation is not clear and cognitive deficits may arise from factors other than seizures or interictal epileptiform discharges. The authors provide evidence for a treatment that may improve cognition and thereby quality of life, raising another possible target of treatment.

Finally, this paper adds to the building evidence that administration of ketone bodies has a direct positive effect, independent of hypoglycemia. Detailed understanding of the multiple mechanisms of dietary treatment may also allow for modification of other existing dietary therapies.

## References

[1] Kim Y, Simeone KA, Simeone TA, Pandya JD, Wilke JC, Ahn Y (2015). Ketone bodies mediate antiseizure effects through mitochondrial permeability transition. Ann Neurol.

[2] Keene DL (2006). A systematic review of the use of the ketogenic diet in childhood epilepsy. Pediatr Neurol.

[3] Rho JM, Sankar R (2008). The ketogenic diet in a pill: is this possible?. Epilepsia.

[4] Beghi M, Cornaggia CM, Frigeni B, Beghi E (2006). Learning disorders in epilepsy. Epilepsia.

